# Wheel running in the wild

**DOI:** 10.1098/rspb.2014.0210

**Published:** 2014-07-07

**Authors:** Johanna H. Meijer, Yuri Robbers

**Affiliations:** Laboratory for Neurophysiology, Department of Molecular Cell Biology, Leiden University Medical Centre, Einthovenweg 20, PO Box 9600, 2300 RC Leiden, The Netherlands

**Keywords:** wheel running, nature, exercise, stereotypic behaviour, circadian rhythms

## Abstract

The importance of exercise for health and neurogenesis is becoming increasingly clear. Wheel running is often used in the laboratory for triggering enhanced activity levels, despite the common objection that this behaviour is an artefact of captivity and merely signifies neurosis or stereotypy. If wheel running is indeed caused by captive housing, wild mice are not expected to use a running wheel in nature. This however, to our knowledge, has never been tested. Here, we show that when running wheels are placed in nature, they are frequently used by wild mice, also when no extrinsic reward is provided. Bout lengths of running wheel behaviour in the wild match those for captive mice. This finding falsifies one criterion for stereotypic behaviour, and suggests that running wheel activity is an elective behaviour. In a time when lifestyle in general and lack of exercise in particular are a major cause of disease in the modern world, research into physical activity is of utmost importance. Our findings may help alleviate the main concern regarding the use of running wheels in research on exercise.

## 1. Introduction

Exercise is beneficial for health and protects against cancer [1], diabetes [2], cardiovascular problems [3], sleep disorders [4] and depression [5]. Activity also stimulates neurogenesis [6,7], even in ageing rodents, in, for example, the hypothalamus and dentate gyrus. Voluntary wheel running is therefore used in many scientific disciplines as a tool to stimulate and measure activity [1–9]. The biological significance of wheel running remains elusive, however, and difficult to interpret [10]. Wheel running is claimed to be unnatural, possibly even a stereotypy or neurosis that develops only in captivity [10,11]. The closest to a formal experiment is a personal communication from Konrad Lorenz, cited by Kavanau [12], which mentions that escaped rodents that were previously exposed to wheels will enter and run in accessible running wheels.

**Figure 1.**
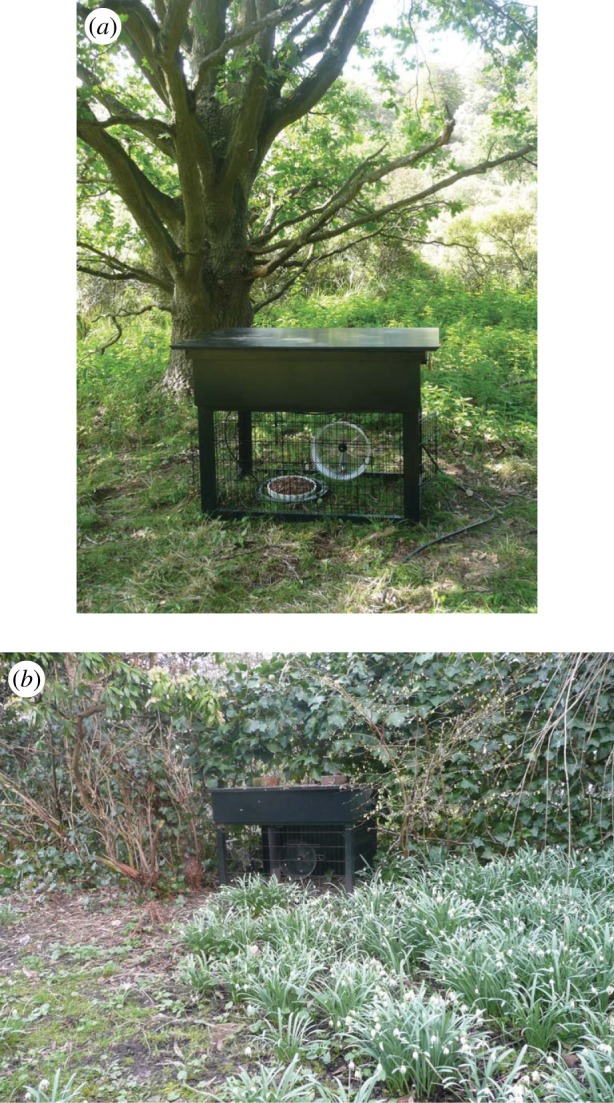
Photographs of the experimental set-up. The set-up is shown *in situ* in the (*a*) dunes and (*b*) urban area. Note that even though the set-up resembles a cage, any animal that is not larger than a rat can freely enter and exit the recording area, food tray and wheel.

The use of running wheels in laboratory experiments is increasing owing to the reported positive impact of exercise on health, and its protective effects on the development of disease [13,14]. This development in laboratory studies reiterates the question whether running wheel activity is a pathological phenomenon that develops only in captivity. Consequently, we have undertaken a study in which we inquired whether running wheel activity is also expressed in the wild, by free-living animals encountering a wheel in their natural habitat. We selected two locations where feral mice live: a spacious, green urban area (data collected October 2009–February 2013) and a dune area not accessible to the public (data collected June 2011–January 2013). In each of these locations, we placed a running wheel (diameter 24 cm) with automatic movement detection, a passive infrared motion sensor, a camera with night vision and a food tray to attract mice. In order to accommodate the measuring equipment, the wheel was part of a cage-like construction that could be easily entered by any animal up to the size of a rat (figure 1*a,b*). Every visit of an animal to the experimental set-up was recorded by the camera, using passive infrared motion detection. At night, active infrared light enabled the camera to work. Infrared light is invisible to mice [15,16] and did not hinder motion detection. The footage recorded made it possible to identify the species responsible for movement of the wheel. Over a period of over 3 years, we have analysed more than 12 000 video fragments in which wheel movement was detected, out of more than 200 000 recordings made when animals visited the recording site. Video recordings were analysed by trained observers in order to determine the species of the animal in the wheel. Wheel movement was detected automatically using a small magnet attached to the wheel and a stationary magnosensor. Wheel running was scored when the sensor was activated.

## 2. Observations in nature

**Figure 2.**
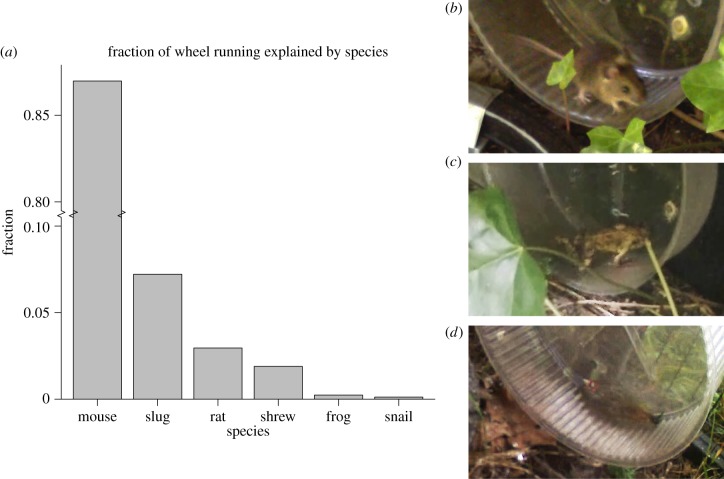
Various animals use the running wheels, though mice are by far the most common. A breakdown by species is given in (*a*). Please note that the vertical axis has been broken in order to accommodate the mice, which accounted for 88% of the wheel running. Also note that birds visited the recording equipment occasionally, but never ran in wheels. Species were identified using video recordings. Stills taken from these recordings show examples of (*b*) a mouse, (*c*) a frog and (*d*) a slug using the wheel.

We observed wheel running both in the urban area (1011 observations in the first 24 months, of which 734 were of mice) and in the dunes (254 observations in 20 months, of which 232 were of mice). Wheel movement not caused by mice was caused by shrews, rats, snails, slugs or frogs (figure 2 and the electronic supplementary material, movie clips). Of these, only the snails caused haphazard rather than directional movement of the wheel and were therefore excluded from the analysis. Cases where animals set the wheel in motion from the outside were also not considered proper wheel running and were therefore excluded.

**Figure 3.**
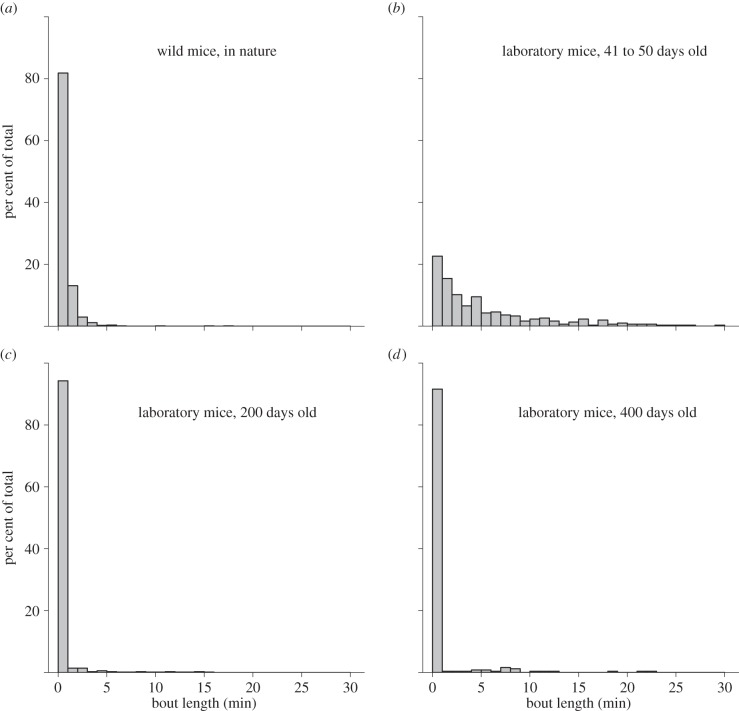
Distribution of running wheel bout lengths in 1 min bins. Results are shown for (*a*) the urban area, (*b*) juvenile mice in the laboratory, (*c*) 200 day old mice in the laboratory, and (*d*) 400 day old mice in the laboratory. Note that while juvenile mice in the laboratory have higher median bout lengths than their wild counterparts, this difference disappears when mice grow older. Mice of 200 days old run as much as the ones in nature, while older mice run less. Data in panels (*b–d*) based on results from [12] with permission.

The observations showed that feral mice ran in the wheels year-round, steadily increasing in late spring and peaking in summer in the green urban area, while increasing in mid-to-late summer in the dunes, reaching a peak late in autumn (see the electronic supplementary material, figure S1*a,b*). Some animals seem to use the wheel unintentionally, but mice and some shrews, rats and frogs were seen to leave the wheel and then enter it again within minutes in order to continue wheel running. This observation indicates that wheel running may well be intentional rather than unintentional for these animals. Video recordings show that the wheel running mice were primarily juveniles, possibly explaining the higher incidence of wheel running around the summer. Mice ran for more than 1 min in 20% of the cases, with a maximum duration of 18 min (figure 3). When compared with running in the laboratory, this is similar to what 200 day old C57BL6 mice do [17]. The mice only ran in our wheels and never walked slowly. The median running speed of mice in the field is less than that of mice in the laboratory (1.3 versus 2.3 km h^−1^; Mann–Whitney *U*-test, *p* < 0.0001), although the maximum running speed observed in the field is higher than the maximum found in the laboratory (5.7 versus 5.1 km h^−1^). Running speed was determined for each bout by multiplying the number of revolutions of the wheel in that bout by the wheel circumference, and dividing by the bout length. A comparison between the laboratory and the field with regard to the covered running distance is complicated by the fact that in the field running occurs mostly outside the wheel [10,18]. Wheel running distance in the laboratory is also highly variable, depending on, for example, age of the animal, diameter of the wheel, rotational friction of the wheel and cage size [10,18,19]. The total reported running distances in nature overlap with the range of distances observed in the laboratory [17,18,20].

We next analysed circadian patterns in wheel running. In absolute number of bouts, there is more wheel running during the night than during the day both in the dunes and the urban area (Mann–Whitney *U*-test, 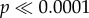 in both cases). Because most visits occurred during the night, we determined the fraction of daily and nightly visits to the recording set-up that included wheel running. In the dunes, a significantly higher fraction of visits to the experimental set-up include wheel running during the night than during the day (Mann–Whitney *U*-test, 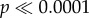). In the urban area, however, the fractions of visits with wheel running were not significantly different between day and night (Mann–Whitney *U*-test, *p* = 0.1602), which was a surprising finding. As low levels of light intensity during the dark phase are known to disturb rhythmicity in mice and rats [21–25], these data may indicate that light pollution during the night affects animal behaviour in urban areas.

## 3. Food as a reward?

One might question whether it was the presence of food near the running wheels that induced animals to run. In order to test whether mice would still run in wheels when no food is present, we stopped providing food in the urban area for more than a year (data collected October 2011–February 2013). We observed that wheel running continued (78 observations of wheel running: 62 of mice; 36 of these mice were still very small, indicating they were too young to have experienced the presence of food). The number of visits to the site dropped significantly as soon as the food was taken away (Mann–Whitney *U*-test, 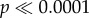). Expressed as a fraction of all visits to the recording area, however, wheel running activity had increased by 42% (Mann–Whitney *U*-test, *p* = 0.0034). The peak in late autumn remained, though a secondary peak in spring emerged (see the electronic supplementary material, figure S1*c*). As in the situation with food present, there was no significant difference in the fraction of day-time and night-time visits that led to wheel running (Mann–Whitney *U*-test, *p* = 0.6558). The continuation of wheel running in the absence of food indicates that wheel running was not triggered by the presence of a rewarding stimulus in the near environment.

## 4. A better understanding of wheel running

There is still much debate over the question whether wheel running is or is not stereotypic [8,9,10,12,26–28]. Stereotypic behaviour itself is characterized by several traits: it is repetitive, invariant and devoid of obvious goal or function [26,27]; if it consists of natural behavioural elements, these occur at higher rates and for longer durations than found in nature [8] and it is partially or not at all dependent on external stimuli [28,29]. Even though authors disagree over whether stereotypies reflect bad welfare [30–33] or a coping strategy that may even increase welfare [27,32], they all agree that stereotypic behaviour only occurs in captivity. Wheel running can be considered repetitive, invariant, devoid of obvious goal and function [8,10], but it remains reactive to external stimuli [10], and our results indicate that it is neither restricted to captivity nor occurring for longer durations in captive mice of at least six months old than in free-ranging mice in the field. Therefore, it does not fit well within the definition of stereotypy.

Given that wheel running can occur as a voluntary behaviour, the question remains why animals choose to run in wheels. A predominant view is that more than one factor determines wheel running [1,10,34,35]. Existing explanations are that wheel running is a consummatory behaviour satisfying a motivation such as play or escape [10], or that it is linked to the metabolic system as a motor response to hunger or to external stimuli relating to foraging [34,35]. Our results indicate that while the number of visits to the recording site decreased when no food was present, the fraction of visits including wheel running increased. This implies that wheel running can be experienced as rewarding even without an associated food reward, suggesting the importance of motivational systems unrelated to foraging. The importance of research into physical activity is increasing. In the modern world, lifestyle is responsible for almost two-thirds of all cases of disease globally [14,36], and the World Health Organization has pinpointed lack of exercise as one of the most important factors causing lifestyle diseases [14,36]. Research into health effects of exercise depends on the use of running wheels [35], and for such research, it would be potentially problematic if running wheel behaviour is stereotypic rather than elective. Our study indicates that running in wheels can be a voluntary behaviour for feral animals in nature.

## Notes

All our experiments are evaluated by the Animal Experiment Committee of Leiden University Medical Centre and performed only after receiving their explicit permission. Since these experiments consisted solely of passive registration of the behaviour of free-living animals in the wild, no permits were required.
